# Interactions of the Intestinal Epithelium with the Pathogen and the Indigenous Microbiota: A Three-Way Crosstalk

**DOI:** 10.1155/2008/626827

**Published:** 2008-10-29

**Authors:** C. V. Srikanth, Beth A. McCormick

**Affiliations:** ^1^Department of Pediatric Gastroenterology and Nutrition, Mucosal Immunology Laboratories, Massachusetts General Hospital, Charlestown, MA 02129, USA; ^2^Department of Microbiology and Molecular Genetics, Harvard Medical School, Boston, MA 02115, USA

## Abstract

The mucosal surfaces of the gastrointestinal tract harbor a vast number of commensal microbiota that have coevolved with the host, and in addition display one of the most complex relationships with the host. This relationship affects several important aspects of the biology of the host including the synthesis of nutrients, protection against infection, and the development of the immune system. On the other hand, despite the existence of several lines of mucosal defense mechanisms, pathogenic organisms such as Shigella and Salmonella have evolved sophisticated virulence strategies for breaching these barriers. The constant challenge from these pathogens and the attempts by the host to counter them set up a dynamic equilibrium of cellular and molecular crosstalk. Even slight perturbations in this equilibrium may be detrimental to the host leading to severe bacterial infection or even autoimmune diseases like inflammatory bowel disease. Several experimental model systems, including germ-free mice and antibiotic-treated mice, have been used by various researchers to study this complex relationship. Although it is only the beginning, it promises to be an exciting era in the study of these host-microbe relationships.

## 1. INTRODUCTION

A mature human gut harbors a vast number of bacterial residents referred to as the commensal microflora or more recently as “microbiota.” It has been estimated that this microbiota is made up of more than 10^14^ individual bacteria comprising over 500 different species [1]. Notably, the composition of the microbiota is individual specific and the type of species residing in the gastrointestinal (GI) tract varies with the host organism's age, diet, and health status [2]. In fact, the total number of microbes in the human GI tract far exceeds (>10–100 times) the sum of all our somatic and germ cells. The biological outcome of this vast and complex population of microbes is that their genes (termed the microbiome) synthesize about 100 times more proteins than the somatic cells of their host [3].

Not surprisingly, the human intestine is more densely populated with microorganisms than any other organ and is a site where they exert a strong influence on human biology. This is because the intestinal mucosa serves as the primary border between the immune system and the external environment, and in addition plays a central role in host-commensal flora interactions. Accumulating evidence indicates that the gut microbiota is instrumental in supporting energy metabolism and immune function of the host. More recent studies suggest that the commensal microbiota play an important role in the development of numerous conditions, including obesity [4, 5], diabetes [6], nonalcoholic fatty liver disease [7], inflammatory bowel disease [8], and perhaps cancer [9]. Unfortunately, the immense complexity of gut flora together with its highly complicated interactions with intestinal epithelium makes it a recalcitrant system to study. Although largely unexplored, our gut microbiota plays an intricate and under-appreciated pivotal role for our health and well-being. In this review we will discuss new developments in the field that highlight the cellular and molecular basis of the crosstalk between the host, the commensal microbiota, and pathogenic bacteria in a healthy as well as a diseased GI tract.

## 2. ROLE OF THE MICROBIOTA IN THE GASTROINTESTINAL TRACT

The microflora of the intestinal microenvironment as a unit provides important protective, metabolic, and trophic functions. Resident bacteria serve a central line of resistance to colonization by exogenous microbes, and thus assist in preventing the potential invasion of the intestinal mucosa by an incoming pathogen. This protective function is known as the barrier effect or colonization resistance and serves a number of important roles. For instance, adherent nonpathogenic bacteria can often prevent attachment and subsequent entry of suspected pathogens into epithelial cells, as well as compete for nutrient availability. The commensal microbiota also helps maintain GI nutrient homeostasis by administering and consuming all resources. For example, dietary nutrients are absorbed by the gut and together with various nonnutrient compounds produced by the microbiota are cometabolize by host enzymes, such as cytochrome P450 and conjugating enzymes in the liver [10]. The resulting metabolites that are derived from both host and microbial processes are returned to the gut by the bile for further metabolism or excretion [11]. This mutual and beneficial relationship helps to dampen unwanted overproduction of nutrients, which could potentially support intrusion of microbial competitors with a potential pathogenic outcome for the host [12].

Quite remarkably, an absence of intestinal bacteria is associated with reduction in mucosal cell turnover, vascularity, muscle wall thickness, motility, baseline cytokine production, digestive enzyme activity, and defective cell-mediated immunity [13]. Indeed, comparative studies in germ-free and conventional animals have established that the intestinal microflora is essential for the development and function of the mucosal immune system during early life, a process that is now known to be important to overall immunity in adults. For example, it has been well established that the number of intraepithelial and lamina propria T cells is lower in germ-free animals, a feature that is reversed upon the restoration of the normal flora [14]. Likewise, levels of secretory IgA are low in the intestine of germ-free animals but are markedly increased upon intestinal colonization of the commensal bacterium, *Bacteroides thetaiotamicron* [15]. Furthermore, the intimate relationship between the commensal microbiota and the intestinal epithelium are involved in shaping the memory mechanisms of systemic immunity, such as oral tolerance. This was initially recognized by the discovery that the systemic response to a specific pathogen can be abrogated after ingesting the antigen; this effect continues for several months in conventionally colonized mice, whereas in germ-free mice systemic unresponsiveness persists for only a few days [16]. Therefore, the innate immune system discriminates between potential pathogens from the commensal microbiota by inducing tolerance to microbial epitopes. This, in turn, dampens responses to commonly encountered foodstuffs and other environmental antigens. Collectively, these examples help to illustrate the important concept that the commensal microbiota profoundly influence the development of the gut mucosal immune system and are essential in preventing exogenous pathogen intrusion.

The intestinal microflora also makes important metabolic contributions by producing vitamin K, folate, and short-chain fatty acids (a major energy source for enterocytes), and mediates the breakdown of dietary carcinogens as well [2, 17]. Perhaps the major metabolic function of the colonic microflora is the fermentation of nondigestible carbohydrates. These nondigestible carbohydrates include large polysaccharides (i.e., resistant starches, pectins, cellulose), some oligosaccharides that escape digestion, as well as unabsorbed sugars and alcohols. The primary metabolic endpoint of such fermentation is the generation of short-chain fatty acids (acetate, proprionate, butyrate). A fundamental role of short-chain fatty acids on colonic physiology is their trophic effect on the intestinal epithelium. Therefore, short-chain fatty acids appear to play an essential role in the control of epithelial cell proliferation and differentiation in the colon. Recent studies have also shown effects of butyrate on intestinal barrier function [18]. Moreover, it has been shown that commensal bacterial can modulate gene expression in the host in order to create a sustainable environment for themselves, while at the same time prevent the growth of other competitive bacteria within the intestinal ecosystem [15].

For the host to thrive and produce more gut residents, the gut microbial ecosystem must be functionally stable over time despite the internal dynamics of the community. Constituent bacteria are expected to have a high degree of functional redundancy between species, so that the loss of one lineage does not adversely impact the homeostatic balance of the intestinal microenvironment [19]. While it is unclear how the selective pressures, microbial community dynamics, and the intestinal microenvironment shape the genome and subsequent functions of members of the gut microbiota, there are some exciting new developments in the field. For example, Gordon et al. have introduced the provocative concept that the evolution of the gut microbiome also likely plays a significant role in shaping the evolution of humans [19]. This tenet is founded on experiments in which this team of investigators sequenced the genomes of two gut-dwelling Bacteroidetes and compared their genomes to the genomes of other bacteria that live both inside and outside of the human body. Quite remarkably, they discovered that lateral gene transfer, mobile genetic elements, and gene amplification play an important role in affecting the ability of the Bacteroidetes to vary their cell surface, sense their environment, and harvest nutrient resources present in the distal intestine [19]. Importantly, these findings lay the conceptual groundwork to suggest that adaptation to the gut ecosystem is a dynamic process that includes acquisition of genes from other microorganisms, and further underscores the significance of considering the evolution humans from the perspective of the evolution of the microbiome [19, 20].

## 3. RESTRICTING PATHOGENS AND COMMENSAL FROM INVADING BEYOND THE MUCOSAL SURFACE

**Figure 1:**
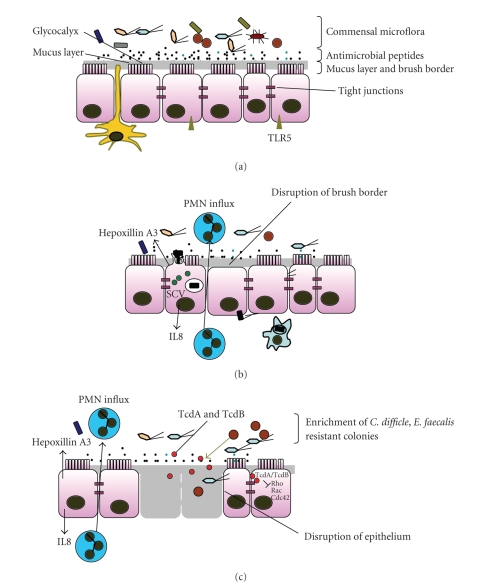
(a) Healthy epithelial surface. A healthy intestinal epithelial surface acts as a physical and biochemical barrier with key features including the apical brush border, the mucus layer, the presence of antimicrobial peptides (blue black dots) in the lumen, the glycocalyx, and the epithelial tight junctions. Also seen in the illustration are numerous commensal bacteria and a dendritic cell sampling the lumen with its extended dendrites (yellow). (b) Key features of *S. typhimurium* infected epithelium. Such host pathogen interactions involve translocation of bacterial effectors (green circles) into the epithelial cells, membrane ruffling, bacterial endocytosis, and SCV formation. Chemoatractants are secreted by the epithelial surface that leads to PMN influx. SCV: Salmonella containing vacuole. (c) Intestinal epithelial surface of an antibiotic-treated patient showing enrichment of a set of antibiotic resistant members of the commensal microflora (light blue and brown) such as *C. difficle* and *E. faecalis*. The *C. difficle* proteins, TcdA and Tcdb (red circles) act intracellularly as glycosyltransferases and inhibit Rho, Rac, and Cdc42. The effect of these modifications lead to actin condensation, transcriptional activation of several genes and apoptosis. Other mechanisms that are triggered include basolateral IL8 secretion, apical Hepoxillin A synthesis, and PMN influx in the apical surface.

The host is protected from potentially harmful enteric microorganisms by the physical and chemical barriers created by the intestinal epithelium that are primarily comprised of absorptive villus enterocytes [21]. The apical surface of the enterocytes are highly differentiated structures consisting of rigid, closely packed microvilli whose membranes contain stalked glycoprotein enzymes [22, 23]. In addition, the tips of enterocyte microvilli are coated with a 400–500 nm thick meshwork referred to as the filamentous brush border glycocalyx [24] and is composed of highly glycosylated transmembrane mucins [25, 26]. The intestinal epithelial barrier is also composed of enteroendocrine cells, goblet cells, and Paneth cells. Microfold (M) cells are also present in the follicle-associated epithelia where they represent a morphologically distinct epithelial cell type whose primary function is in the transport of macromolecules, particles, and microorganisms from the lumen to underlying lymphoid tissue [27, 28]. Intercellular junctional complexes that are composed of tight junctions, adherens junctions, and desomosomes maintain the integrity of the epithelial barrier. The most apical components of the junctional complex are the epithelial tight junctions, which are highly regulated and serve to create a semipermeable diffusion barrier between individual cells (Figure 1(a)). Collectively, these features facilitate the intestinal epithelium to act as a physical barrier to prevent unwanted bacteria from gaining access to the host.

The intestinal epithelium also provides a unique surface that is armed with a bounty of specialized cells that produce mucus, antimicrobial peptides, and antimicrobial molecules, which together form the front line of defense against pathogenic microorganisms (Figure 1(a)). The mucus layer is secreted by the goblet cells and this layer overlies the intestinal epithelium to create a physical blockade against offending enteric microbial pathogens. For example, it has been demonstrated that secreted mucus acts as a barrier to *Yersinia enterocolitica* [29], rhesus rotavirus [30], and *Shigella flexneri* [31]. The commensal microbiota has also been found to regulate the production of intestinal mucins, which consequently inhibits the adherence of numerous pathogenic bacteria to intestinal epithelial cells [32–34]. Paneth cells are another important cell type that are involved in intestinal defense against potential harmful pathogenic bacteria. These cells are present at the base of the crypt of Lieberkühn [35] and have been shown to produce a number of antimicrobial peptides. In addition, the gastrointestinal expression of antimicrobial peptides is evolutionarily conserved [36], and to date, *α*-defensins (HD), *β*-defensins (hBD), and cathelicidins have been identified in humans [37]. Paneth cells also produce a number of antimicrobial molecules, including lysozyme, phospholipase A_2_, and angiogenin-4 (reviewed in [37]). Therefore, it is inferred by numerous studies that Paneth cells are able to control the bacterial ecosystem (Table 1).

Angiogenin-4 is expressed mainly in the small intestine, cecum, and colon and acts on Gram-positive bacteria [49, 50]. However, most antimicrobial peptides expressed by mammalian epithelial cells are members of peptide families that mediate nonoxidative microbial cell killing by phagocytes [50]. These amphipathic molecules interact with and lyse bacterial membranes [55]. Defensins generally possess a broad range of antimicrobial activity (Table 1). In particular, human intestinal defensin-5 has been shown to kill *Listeria monocytogenes*, *E. coli,* and *Candida albicans* [40]. Additional evidence supporting a critical role for defensins in vivo was demonstrated in a study utilizing human defensin-5 transgenic mice; these mice exhibited marked resistance to oral challenge with virulent *Salmonella enterica* serovar Typhimurium (*S. typhimurium*) [39]. The intestinal epithelial cells also express another class of antimicrobial peptide, the cathelicidins (LL-37/Cap18), in which a cathelin domain is linked to a peptide with antimicrobial activity [56]. LL-37 is expressed within the epithelial cells located at the surface and upper crypts of normal human colon. Although little or no expression is seen within the deeper colonic crypts or within epithelial cells of the small intestine, studies in mice have determined these molecules to be protective against bacterial pathogens [47]. Interestingly, the expression of these factors, unlike the angiogenins, is not induced by the presence of pathogenic bacteria but rather their secretion is triggered by the commensal microbiota and/or their derivatives. A recent addition to this growing list of intestinal antimicrobial includes RegIII*γ*, which has been shown to be toxic to Gram-positive bacteria [52]. RegIII*γ* is a C-type lectin that binds to the carbohydrate moiety of bacterial cell wall constituent, petidoglycan. Recent studies have further shown that the expression of RegIII*γ* is strongly dependent upon the presence of the gut microflora since in germ-free mice RegIII*γ* expression is severely repressed [53] (Table 1).

The intestinal epithelium also provides a surface where the host can sense the microbial microenvironment in order to elicit an appropriate defense response by releasing an array of signaling molecules (i.e., chemokines and cytokines). These molecules then trigger the recruitment of leukocytes to initiate an early inflammatory response. Paradoxically, however, although continuously exposed to Gram-positive and Gram-negative bacteria and their products (i.e., lipopolysaccarhide (LPS), peptidoglycan, and lipoprotein) the normal healthy intestinal mucosa maintains a mechanism of hyporesponsiveness to the lumenal microbiota and their products. Exaggerated inflammatory responses in the absence of pathogenic bacteria would be otherwise deleterious [57, 58]. Accordingly, the normal intestinal epithelial host defenses are able to accurately interpret the complex microbial environment in order to discriminate between permanently established commensal microbes and episodic pathogens.

At the core of this strategy the endogenous microbiota all share “self” signature molecules termed microbe-associated molecular patterns [59]. However, upon infection of a pathogenic organism, the host immune response is activated by the specific recognition of “nonself” molecular structures known as pathogen-associated molecular patterns. The epithelial cells are able to sense the microenvironment within the gut by means of pattern recognition receptors (PRRs) that include Toll-like receptors (TLRs) and nucleotide-binding oligimerization domain (NOD) proteins [38, 60–63]. TLRs are evolutionary conserved and are characterized by an extracellular leucine rich repeat (LRR) domain (involved in ligand recognition), as well as an intracellular Toll/interleukin-1 receptor-like domain (involved in proinflammatory signal transduction) [60, 64–66]. In addition, two NOD proteins (NOD1 and NOD2) function as intracellular sensors of bacterial products in the induction of an inflammatory response [60, 64–67].

These PRRs recognize bacterial factors, such as LPS, lipoproteins, flagellin, unmethylated-CpG DNA, and a large number of other specific components. Regulation of the expression and the specific location of TLRs and NODs in intestinal epithelial cells fosters efficient immune recognition of the commensal microflora and maintains a delicate balance; permitting a basal level of signaling events to proceed, while at the same time restraining innate immune responses. For instance in a healthy intestine, epithelial cells express very little or no TLR2, TLR4, and CD14, and as a result minimizes the recognition of commensal LPS [68, 69]. TLR5, which recognizes bacterial flagellin, has been reported to be expressed exclusively on the basolateral surfaces of the epithelial cells. This TLR is ideally positioned to detect its ligand, translocated flagellin [70]. Moreover TLR3, TLR7, TLR8, and TLR9 are expressed in the intracellular endosomal compartments [71]. These intracellular PRRs would not ordinarily encounter luminal commensal bacteria or those attached to the apical surface of intestinal epithelial cells but are well positioned to recognize pathogenic bacteria that actively breach the epithelial barrier. As an additional measure, commensal bacteria have the ability to induce the expression of intestinal alkaline phosphatase, which not only dephosphorylates dietary lipids but also dephosphorylates the LPS of commensal flora resulting in reduced toxicity in mammals [72].

Nonpathogenic microorganisms may also be able to selectively attenuate the NF-kB pathway as mechanism of intestinal immune tolerance. Neish et al. initially reported that colonization of a human model intestinal epithelium with certain strains of nonpathogenic bacteria could dampen the host cell responses to subsequent proinflammatory challenges by blocking the proinflammatory/antiapoptic NF-*κ*B pathway [73]. This effect is mediated by the inhibition of I*κ*B-*α* ubiquitination, which prevents regulated I*κ*B-*α* degradation, NF-*κ*B nuclear translocation, and subsequent activation of proinflammatory/antiapoptic genes. I*κ*B-*α* ubiquitination is catalyzed by E3-SCF^*β*−TrCP^ ubiquitin ligase [74], which is regulated via covalent modification of the cullin-1 subunit by the ubiquitin-like protein NEDD8 [75, 76]. Recently, it was determined that the interaction of nonpathogenic bacteria with epithelial cells results in the rapid loss of neddylated Cul-1 and consequent repression of the NF-*κ*B pathway [77]. Collectively, this set of observations underscores the ability of intestinal bacterial communities to influence eukaryotic processes, and perhaps more specifically demonstrates inflammatory tolerance of the mammalian intestinal epithelia.

## 4. HOW PATHOGENS OVERCOME THE EPITHELIAL BARRIER

As described above, the intestinal epithelium has evolved a rather formidable fortress to guard against microbial invasion. However, through a process of coevolution, potential harmful enteric microorganisms have evolved counter strategies to hijack the cellular molecules and signaling pathways of the host to become potentially pathogenic. As an initial step in the infection process, certain enteric pathogens target specific epithelial cell structures, including glycoproteins and glycolipids, which serve as receptors for bacterial attachment [78]; thus, enabling them to exploit the underlying signal transduction pathway. Other strategies utilized by invading enteric pathogens, such as *S.* *typhimurium* and *Shigella flexneri* have evolved a sophisticated strategythat directs the entry of the enteric pathogen into intestinal epithelial cells. This process requires the expression of a bacterial type III protein secretion system (TTSS), the function of which is to deliver a set of effector proteins into the host cell [79–81]. These effector proteins co-opt host cell signal transduction cascades as a clever means of subverting normal host cell processes by triggering a marked rearrangement of the host cytoskeleton. This entry mechanism termed bacterial mediated endocytosis drives bacterial entry and facilitates the pathogen to cross the epithelial barrier as well as to induce a proinflammatory response [79–81].

The latter step in this process can be achieved by direct cytotoxic injury, intracellular migration, disruption of the epithelial tight junctions, or indirectly by inducing neutrophil infiltration. Although several bacterial pathogens have been able to modulate epithelial tight junctions to their own advantage, the direct interaction of a bacterial virulence factor on component proteins of the tight junction has been proposed only in a few instances [82]. It is well documented that anumber of enteric pathogens perturb the intestinal epithelial barrier and impact TER or paracellular permeability, most often with an alteration in the arrangement of tight junctional component proteins by mechanisms that are unique for different pathogens [82]. For example, *Clostridium difficile* toxins A and B enhance epithelial cell permeability by disrupting actin microfilaments within the perijunctional ring [83], and enteropathogenic *Escherichia coli* disrupt the epithelial barrier by the phosphorylation of myosin light chains [84]. With respect to *S. typhimurium*, in vitro models of infection have revealed an alteration of epithelial permeability and loss of barrier function, which involves rapid changes in both tight junction permeability and transcellular conductance [85, 86]. Recent studies further indicate that the *Salmonella* effector protein SigD (also called SopB), which is encoded in *Salmonella* pathogenicity island-1 (SPI-1), is able to elicit a reduction in epithelial barrier function, perhaps via activation of PKC [87]. Also, the effector proteins SopB, SopE, SopE2, and SipA are necessary to disrupt the epithelial barrier and alter the distribution of at least some tight junction proteins [88, 89]. Such perturbations in the components of the tight junction lead to enhanced bacterial translocation and infiltration of neutrophils across the intestinal barrier. Therefore, the ability to regulate the molecular composition of the tight junctions facilitates the pathogenecity of *S. typhimurium* by fostering its uptake and distribution within the host (Figure 1(b)) [85].

*S. flexneri* has a distinct mode of pathogenesis that involves entry into colonic epithelial cells from the basolateral surface [90], thereby requiring its relocation from the lumenal to the underlying surface of the epithelium. This translocation event has historically been attributed to the uptake and transport by M cells [91]. However, it has since been established that *Shigellae* are also capable of altering components of the tight junctional complex, allowing the bacteria to traverse the paracellular space to reach the basolateral surface; an event that also decreases barrier function [92]. Once at the basolateral surface, *Shigellae* rapidly invade and disseminate through the epithelium, causing a further decrease in barrier function [92–94] through the action of a TTSS system and additional proteins encoded on a large virulence plasmid [94–97].

Enteric pathogens cause a variety of diseases in humans but one undeniable symptom is the presentation of gastroenteritis. Some bacterial enteric infections are characterized by disruption of the normal movement of electrolytes and water across the epithelium, which is converted from a state of net fluid absorption to one of net fluid secretion [98]. Secretory diarrhea, as a result of epithelial chloride secretion, has long been regarded as a host defense mechanism. This is based on the notion that increased fluid and electrolyte movement into the gut lumen helps to inhibit adherence of pathogenic organisms by “flushing” them from the body. However, it could also be argued that the induction of pathogen-induced diarrhea is a way to ensure transmission to new hosts, and thus pathogenic fitness [99]. These ideas are not mutually exclusive and secretory diarrhea may be advantageous to both host and pathogen.

Pathogenic bacteria cause diarrhea by multiple mechanisms. *Vibrio cholerae* reside in the lumen of the small intestine and produce toxins, which alter ion absorption and/or secretion [100, 101]. Other bacteria such as *Shigella* and enteroinvasive *E. coli* invade and destroy the colonic epithelium leading to dysentery [102]. More recently pathogenic *E. coli* have been shown to increase chloride ion secretion from intestinal epithelia by upregulating the expression of the receptor for the neuropeptide galanin 1 [103]. Rotavirus, another important cause of diarrhea in infants, induces this condition by activating the enteric nervous system [104, 105].

A large influx of neutrophils (PMNs) into the mucosa and lumen from the underlying vasculature is a significant feature of intestinal bacterial infections [105, 106]. During infection of epithelial cells by enteric pathogens such as *S. typhimurium* and *S. flexneri*, IL-8 is synthesized and secreted baslaterally. Such basolateral IL-8 release imprints subepithelial matrices with long-lived haptotactic gradients that serve to guide neutrophils through the lamina propria to a subepithelial position [107]. However, basolateral IL-8 release is insufficient to induce the migration of neutrophils across the intestinal epithelium, suggesting that the production of other inflammatory mediators, whose release would probably be polarized apically, is important for the execution of this step in the inflammatory pathway [107, 108]. In support of this contention, Kucharzik et al. recently developed a double transgenic mouse model with the ability to induce human IL-8 expression restricted to the intestinal epithelium [109]. The results from this transgenic model showed that although acute induction of IL-8 in the intestinal epithelium is sufficient to trigger neutrophil recruitment to the lamina propria, additional signals are required for neutrophil transepithelial migration and mucosal tissue injury. Indeed, recent evidence suggests that the eicosanoid, hepoxilin A_3_, is secreted apically and is responsible for the final step of neutrophil transepithelial migration into the gut lumen [110, 111]. This process is quite complex as distinct signaling pathways mediate *S. typhimurium* invasion, induction of CXCL8 secretion, and induction of hepoxilin A_3_ secretion [111–113].

The ability of *Salmonella* serotypes to elicit PMN transmigration in vitro correlates with their ability to cause diffuse enteritis (defined histologically as transepithelial migration of neutrophils), but not typhoid fever in humans [114]. Moreover, large-scale PMN transepithelial migration causes decreased barrier function [115]. Studies exploring the mechanism underlying the release of HXA_3_ during infection with *S. typhimurium* revealed the involvement of the *S. typhimurium* type III secreted effector protein, SipA [116]. The *S. typhimurium* effector protein, SipA, promotes a lipid signal transduction cascade that recruits an ADP-ribosylation factor 6 guanine nucleotide exchange factor (such as ARNO) to the apical plasma membrane. ARNO facilitates ADP-ribosylation factor 6 activation at the apical membrane, which in turn, stimulates phospholipase D recruitment to and activity at this site. The phospholipase D product, phosphatidic acid, is metabolized by a phosphohydrolase into diacylglycerol, which recruits cytosolic protein kinase C (PKC)-alpha to the apical membrane. Through a process that is less understood, activated PKC-alpha phosphorylates downstream targets that are responsible for the production and apical release of HXA_3_, which drives transepithelial neutrophil movement [117].

## 5. PROTOTYPICAL INTERACTIONS BETWEEN PATHOGENIC BACTERIA AND COMMENSAL MICROBIOTA

There are ample lines of evidence to support the emerging concept that a change in the composition of the commensal microbiota alters the intestinal microenvironment making this niche vulnerable to pathogenic insult. In this section we discuss examples to illustrate the remarkable crosstalk between the host, its intestinal microbiota, and potential pathogenic bacteria.

It has been well documented that *S. typhimurium* causes a systemic (typhoid fever) infection in mice while in humans this enteric pathogen causes gastroenteritis. However, Barthel et al. discovered that pretreatment of C57BL/6 mice with streptomycin, an antibiotic that kills facultative anaerobes, followed by infection with a streptomycin-resistant strain of *S.* *typhimurium* produced a robust intestinal inflammatory response [118]. Such enteritis is primarily characterized by inflammation in the cecum, and also presents with several of the typical pathological hallmarks of acute *Salmonella*-induced gastroenteritis in humans, including PMN infiltration and epithelial cell erosion. This is an intriguing result since the only difference between the untreated and streptomycin treated mice is the alteration of the commensal flora; thus, demonstrating that the presence of the microflora plays a protective role against pathogenic invaders. This study also substantiates the long-standing finding of Barrow and Tucker who found that pretreatment of a chicken's cecum with three different strains of *E. coli* significantly reduced infection with *Salmonella* as compared to untreated animals [119]. Additionally, Hudault et al. (2001) determined that the presence of a single species of *E. coli* in the gut could restrict the infection of S *. typhimurium* as compared to its germ-free counterpart [120].

More recently, Stecher et al. used the *S. typhimurium* colitis model to investigate competition between an enteric pathogen and the host microbiota [121]. This group found that inflammatory responses induced by *S. typhimurium* led to profound perturbations in the composition of the commensal microbiota as determined by 16S rRNA. The inflammatory host responses induced by *S. typhimurium* not only changed the microbiota composition but also suppressed its growth, thereby, overcoming colonization resistance. In contrast, an avirulent *Salmonella* mutant defective in triggering inflammation was unable to overcome colonization resistance. These results raise an interesting point in that perhaps the intestinal inflammation induced by *S. typhimurium* might be a crucial event in order to overcome colonization resistance. In this respect, triggering the host's immune defense may shift the balance between the protective microbiota and the pathogen to favor the pathogen. The idea that the intestinal microbiota can be altered by invading pathogens is further supported by Lupp et al. who found that host-mediated inflammation in response to an infectious agent induced alterations in the colonic community that not only resulted in the elimination of a subset of indigenous microbiota but also led to the growth of the Enterobacteriaceae family [122]. Moreover, in children undergoing treatment for diarrhea, fluctuations in the intestinal microflora were observed for both rotaviral and nonrotaviral-induced diarrhea [123]. This phenotype was reversed and the normal microflora was re-established after about three months of the disease episode. Other studies have investigated the role of the intestinal microbiota during infectious disease transmission. In particular, Lawley et al. describe a model in which persistently infected 129X1/SvJ mice provide a natural model of transmission. In this model, only a subset of mice termed “supershedders” could shed high levels of bacteria in their feces. Whereas immunosuppression of the infected mice did not induce the supershedder phenotype, antibiotic treated mice displayed a high supershedder phenotype [124]. Together, these studies suggest that the intestinal microbiota plays a critical role in controlling pathogen infection, disease, and even transmissibility.

There are also examples in which members of the commensal microflora are able to cause disease. This is specifically illustrated by *Enterococcus faecalis*, a prominent member of the GI tract microbiota. In a healthy intestine these bacteria behave as a normal resident of the intestinal ecosystem. However, in individuals undergoing antibiotic treatment or those who are immunocompromised, *E. faecalis* is able to colonize new niches of the intestinal microenvironment as a certain subgroup of this species is antibiotic resistant (Figure 1(c)). Under such compromised conditions, *E. faecalis* can infect and spread to other sites of the host such as the bloodstream, urinary tract, and surgical wounds. Not surprisingly, the subgroup population harboring the antibiotic resistance genes also has genetic elements conferring infectivity and virulence. Furthermore, the genome sequence of *E. faecalis* strain V583, the most causative agent of vancomycin resistant enterococcal infection in America, [125] was recently reported [126]. Recent studies have determined that more than 25% of the *E. faecalis* genome is most likely derived from mobile or foreign DNA, which might have contributed to the rapid acquisition and dissemination of drug resistant strains [126]. Another example is illustrated by *Clostridium* *difficle*, a Gram-positive bacterium that can harmlessly inhabit the human intestine. However, certain individuals undergoing antibiotic therapy, as a result of their altered intestinal microflora, presented with *C. difficle* infection accompanied with severe intestinal colitis (Figure 1(c)) [127].

Commensal bacteria, such as *Bacteroides fragilis*, may also inhibit other opportunistic members of the intestinal microflora from causing disease [128]. *B. fragilis* is a Gram-negative bacterium that resides in a healthy human intestine. Normally, this bacterium expresses a surface carbohydrate capsule known as polysaccharide A (PSA), which contributes to many beneficial activities underlying the immune development of the host, including activation of CD4+ T cells, and stimulation of the innate immune responses through TLR2 signaling. Mazmanian et al. determined that *B. fragilis* protects the host from *Helicobacter hepaticus*-induced colitis in experimental mice. However, in animals harboring *B. fragilis* strains that do not express PSA, *H. hepaticus* colonization led to disease and production of proinflammatory cytokines induced by intestinal immune cells [128, 129]. Thus, in healthy individuals it appears that PSA from *B. fragilis* is necessary to confer some beneficial activity. In spite of this, PSA was also found to potentiate the ability of *B. fragilis* to cause disease in patients who have a compromised mucosal surface, such as postsurgical patients. This function is initiated upon submucosal entry of the bacteria during which PSA activates CD4+ T cells leading to abscess formation [130].

## 6. ROLE OF BACTERIA IN INFLAMMATORY BOWEL DISEASE

Recent evidence from a variety of investigative avenues implicates abnormal host-microbial interactions in the pathogenesis of inflammatory bowel disease (IBD). In fact, IBDs preferentially occur in the colon and distal ileum (i.e., locations that contain the highest concentrations of intestinal bacteria). An important role for microbial agents in the pathogenesis of IBD is inferred by numerous recent studies, which conclude the bacterial flora differs between patients with inflammatory bowel disease (IBD) and healthy individuals. Moreover, accumulating evidence suggests that the composition and function of the microbiota in patients suffering with IBD are abnormal.

Ninety-nine percent of the gut microbiota in healthy individuals is composed of species within four bacterial divisions: *Firmicutes*, *Bacteroidetes*, *Proeobacteia*, and *Actinobacteria* [131, 132]. Investigation of the microbial diversity in active IBD is a highly pursued topic of interest and is an area of research still at its infancy. In IBD patients, early returns have suggested that there is a decrease in the number of beneficial bacteria, such as *Bifidobacterium* and *Lactobacillus* spp., and an increase in pathogenic bacteria, such as a *Bacteroides* and *Escherichia* *coli* [132–136]. Such dysbiosis induces a breakdown in the balance between putative spp. of protective versus harmful bacteria, and may promote inflammation. Other studies have shown that there is a decrease in microbial diversity that accompanies the increased numbers of Enterobacteriaceae, including *E.* *coli*, with decreased numbers of *Firmicutes*, and a particular decrease in *Clostridium* species. As convincing as this data is, there is still a lack of evidence to denote whether a specific pathogen is responsible for onsets or relapses of IBD [132]. Further, the most compelling studies are derived from animal models. Regardless, a number of organisms have been implicated in Crohn's disease, with *Mycobacterium paratuberculosis* and *E. coli* drawing a great deal of attention [137].

Patients with IBD have higher numbers of mucosa-associated bacteria than control patients [138], and the generalized or local dysbiosis observed is due to the presence of low numbers of normal bacteria and high numbers of unusual bacteria with a decrease in biodiversity. The composition of the increased numbers of bacteria attached to the intestinal epithelium of IBD patients are from diverse genera. *Bacteroides spp*., in particular, has been identified as a predominate member of the epithelial layer, and in some instances was located intracellularly [136]. While this remains an intriguing observation, the role of *Bacteroides* in IBD is still unclear. Furthermore, distinct adherent or invasive *E. coli* has been identified in the ileal mucus of patients with Crohn's disease, and the involvement of a new potentially pathogenic group of adherent invasive *E. coli* (AIEC) has been suggested [139]. For instance, in studies aimed to assess the predominance of *E. coli* strains associated with the ileal mucosa of Crohn's disease patients, *E. coli* was recovered from 65% of chronic lesions and from 100% of the biopsies of early lesions. By comparison, 3–6% of the *E. coli* was recovered form healthy ileal mucosa. *E. coli* was also abnormally present (50–100% of the total number of aerobes and anaerobes) in early and chronic ileal lesions of CD patients [140, 141]. These observations were confirmed in a subsequent study in which adherent *E. coli* was found in 38% of patients with active ileal Crohn's disease [133]. This study also revealed that the number of *E. coli* in situ correlated with the severity of the disease, and that the invasive *E. coli* was also restricted to the inflamed mucosa. Interestingly, the recovered *E. coli* strains were predominantly novel in phylogeny, displayed pathogen-like behavior in vitro, and expressed virulence factors [133].

It is suspected that the abnormal colonization of the lieal mucosa is largely due to increased expression of CEACAM6, a receptor for adherent-invasive *E. coli* [142]. However, Crohn's disease patients also exhibit defective microbial killing mechanisms that result in increased exposure to commensal bacteria. For example, Crohn's disease patients have defective antimicrobial peptide production, including *α*-defensin 5 in ileal disease and human *β*-defensin 2 in Crohn's colitis [143, 144]. This is accompanied by functional abnormalities in the killing of *Bacteroides vulgatus*, *E. coli*, and *Enterococcus faecalis* [145]. In addition, NOD2 polymorphisms in Crohn's disease are associated with selective decrease in *α*-defensin production by Paneth cells, as well as in defective clearance of intracellular pathogens by colonic epithelial cells [146]. Thus, combined with defective antimicrobial peptide function in Crohn's disease the functional changes described above provide a reasonable rationale for the profound increase in mucosally associated Enterobacteriaceae. Also, in light of the alteration in the composition of the luminal microbiota, it is perhaps not surprising that Crohn's disease has features that might be the consequence of a microbial process. This is exemplified by the noted infection of Peyer's patches and lymphoid aggregates, and the presence of ulcerations, microabscesses, fissures, fistulas, granulomas, and lymphangitis [137].

As evidence accumulates to suggest that dysbiosis in IBD patients induces a breakdown in the balance between putative spp. of protective versus harmful bacteria, one potential new method of intervention lies in the modulation of the enteric flora. Indeed, current studies suggest that probiotics might offer an alternative or adjuvant approach to conventional IBD therapies by altering the intestinal microflora and, in turn, modulating the host immune system. Probiotics are defined as living food supplements or components of bacteria that have a beneficial effect on human health. Indeed, probiotic activity has been associated with *Lactobacillus*, *Bifidobacteria*, *Streptococcus*, *Enterococcus*, nonpathogenic *E. coli*, and *Saccharomyces bourlardii* [147, 148].

Probiotic supplements may balance the indigenous microflora in IBD patients. A growing body of literature supports this emerging concept, which suggests that probiotics have therapeutic effects in ulcerative colitis, Crohn's disease and pouchitis [147, 148]. The rationale for employing probiotics in the treatment of IBD is underscored by the proposed pathogenic role of the intestinal microflora in this disease. Numerous studies support the notion that introduction of probiotics to the GI tract can alter the enteric microflora in IBD patients, which in turn has a profound effect on intestinal defense mechanisms, including (i) inhibiting microbial pathogenic growth, (ii) increasing epithelial cell tight junctions and permeability, (iii) modulating the immune response of the intestinal mucosa, (iv) increasing the secretion of antimicrobial products, and (iv) eliminating pathogenic antigens [149–151]. Thus, such broad mechanistic effects of probiotics may explain the beneficial effects observed.

Probiotic preparations are primarily based on a variety of lactic acid bacteria (lactobacilli, bifidobacteria, and streptococci), which under healthy conditions are normal and important components of the commensal microbiota. In addition, probiotic mixtures often contain some nonpathogenic bacteria that include *E. coli*, enterococci, or yeast (*Saccharomyces bourlardii*) [152]. Probiotic strains also need to satisfy important criteria. First, probiotics must be safe and tested for human use [149, 152]. In addition, such strains should be of human origin, resistant to acid and bile, and survive and be metabolically active within the intestinal lumen. Probiotics must also be antagonistic against pathogenic bacteria as they produce antimicrobial substances, compete within the GI tract, and promote a reduction in colonic pH.

Many clinical trials have documented that probiotics can achieve and maintain remission in patients with ulcerative colitis, and also prevent and maintain remission of pouchitis. However, probiotics seem to be ineffective in Crohn's disease [153]. Although controlled clinical trials are still required to investigate the unresolved issues related to efficacy, dose, duration of use, single or multistrain formulation, and simultaneous use of probiotics, synbiotics, or antibiotics, the preliminary data for the therapeutic use of probiotics in selective patients with mild to moderate IBD are encouraging.

## Figures and Tables

**Table 1:** Antimicrobial peptides/proteins and their targets.

Class	Examples	Expression	Action	References
*α*-defensin	HD-5, HD-6	Paneth cells	*L. monocytogenes*	[38–41]
			*E. coli*	
			*S. typhimurium*	
*β*-defensin	hBD-1	IECs	*P. aeruginosa*	[42–46]
	hBD-2		*E. coli*	
			*Candida albicans*	
Cathelicidin	hLL37	IECs	*Salmonella*	[47, 48]
Angiogenin	Angiogenin-4	Paneth cells	Gram positive	[49–51]
			Bacteria	
C-type lectin	RegIII*γ*	IECs	Gram positive	[52–54]
			Bacteria	
